# Role of magnetic resonance imaging versus ultrasound for detection of plantar plate tear

**DOI:** 10.1186/s13018-016-0507-6

**Published:** 2017-01-21

**Authors:** Xin Duan, Lang Li, Dai-Qing Wei, Ming Liu, Xi Yu, Zhao Xu, Ye Long, Zhou Xiang

**Affiliations:** 1 Orthopedics Department, West China Hospital, Sichuan University, #37 Guoxuexiang Street, Chengdu, 610017 Sichuan Province China; 2 Department of Bone and Joint Surgery, Affiliated Hospital of Westsouth Medical University, #25 Taiping Steeet, Luzhou, Sichuan Province China; 3 Anesthesia Department, West China Hospital, Sichuan University, # 37 Guoxuexiang Street, Chengdu, Sichuan province China

**Keywords:** MRI, Ultrasonic sound, Plantar plate tear, Diagnosis

## Abstract

**Background:**

Plantar plate tears could be the reason of forefoot pain, affecting foot function. Magnetic resonance imaging (MRI) and ultrasound (US) were commonly used for the diagnosis of plantar plate tears. The decision of whether to use MRI or US carried some controversy. Our study aimed to find out the diagnostic accuracy of MRI versus US for plantar plate tears.

**Methods:**

The database of the Cochrane Central Register of Controlled Trials (CENTRAL), PubMed, EMBASE, and relative orthopedic meetings until May 2016 were searched. Studies involved in the diagnostic detection of MRI or ultrasound for plantar plate tears with surgical criteria as the reference test were included. Data was analyzed by meta-analysis. We compared sensitivity, specificity, positive likelihood ratio, negative likelihood ratio, and summary receiver operating characteristic (sROC) plot of both MRI and US.

**Results:**

Seven studies involving 246 plantar plate tears were included. The MRI showed more diagnostic accuracy than US for the detection of plantar plate tears. The sensitivity, specificity, positive likelihood ratio, and negative likelihood ratio of MRI were 95%, 54%, 2.08, and 0.08, respectively, while the same values for US were 93%, 33%, 1.20, and 0.35, respectively. And the sROC showed more superior diagnostic accuracy than the US.

**Conclusion:**

The current result suggests that MRI has better accuracy than US for detection of plantar plate tears.

## Background

Plantar plates which are made of fibrocartilaginous structures were found in the metatarsophalangeal (MTP) joints. The average length, width, and thickness of plantar plates are 16, 9, and 1.8 mm, respectively [1, 2]. It is located in the central of plantar aspect of the lesser MTP, with the function of supporting the body weight and restricting dorsiflexion [3]. The plantar plate attached to the major longitudinal bands of the plantar fascia, proximal phalanx, and collateral ligaments play an active role in the ankle [1]. Plantar plate tears can be the reason of forefoot pain, affecting foot function and gait [4, 5]. Instability of the MTP joint caused by plantar plate tears has been widely reported and has frequently occurred in uncomfortable footwear [6–8].

The diagnosis of instability is based on clinics but could be improved by imaging studies [2]. Magnetic resonance imaging (MRI) and ultrasound (US) have been commonly used to detect plantar plate tear previously. MRI is a noninvasive method compared to arthrography for the detection of plantar plate and helped to identify intra-articular and extra-articular diagnoses [3, 7]. MRI is frequently used to assess this structure. However, MRI is expensive machine and not every hospital has the MRI. Meanwhile, MRI could not be used in some conditions, such as implant of cardiac pacemakers or automatic defibrillators. US is an available way to assess some disease by pulsed ultrasonic waves. The use of diagnostic ultrasound in the identification of plantar plate tears has been increasingly reported [9, 10]. Diagnostic ultrasound is easy to use and provide a relatively inexpensive option compared with other advanced imaging modalities. However, the US needed skillful operator lead to its limitation [11]. Therefore, the decision of whether to use MRI or US carried some controversy. This study aims to determine the diagnostic accuracy test of MRI versus US for plantar plate tears.

## Review

### Search strategy

This study adhered to the Preferred Reporting Items for Systematic Reviews and Meta-Analyses (PRISMA) guidelines [12]. Any original study, published in a peer reviewed journal, which accessed the diagnostic test accuracy (sensitivity, specificity, positive likelihood ratio, and negative likelihood ratio) of MRI or ultrasound for the detection of adults (>18 years old) with suspect plantar plate tears was included. Surgery or arthroscopy as reference test was included. Studies assessing cadaveric, animal models, pediatric patients, or without diagnostic test accuracy were excluded.

An online searching was conducted for this studies and no language limitation. The Cochrane Central Register of Controlled Trials (CENTRAL), PubMed, EMBASE (to May 2016), and relative orthopedic meetings were searched. The search strategy is shown in Table 1. For the studies reported in duplicate, only the latest or complete reports were collected.

**Table 1:** Search strategy

CENTRAL	PubMed	EMBASE
#1 MeSH descriptor plantar plate, this term only #2 (* planta*) :ti,ab,kw #3 (#1 OR #2) #4 MeSH descriptor MRI, this term only #5 MeSH descriptor magnetic resonance imaging, #6 MeSH descriptor US, this term only #7 MeSH descriptor ultrasonography #8 (#4 OR #5 OR #6 OR #7) #9 (#3 AND #8)	(plantar[All Fields] AND (“bone plates”[MeSH Terms] OR (“bone”[All Fields] AND “plates”[All Fields]) OR “bone plates”[All Fields] OR “plate”[All Fields]) AND (“magnetic resonance imaging”[MeSH Terms] OR (“magnetic”[All Fields] AND “resonance”[All Fields] AND “imaging”[All Fields]) OR “magnetic resonance imaging”[All Fields] OR “mri”[All Fields])) OR (plantar[All Fields] AND (“bone plates”[MeSH Terms] OR (“bone“[All Fields] AND “plates“[All Fields]) OR “bone plates”[All Fields] OR “plate”[All Fields]) AND (“ultrasonography”[Subheading] OR “ultrasonography“[All Fields] OR “ultrasound”[All Fields] OR “ultrasonography”[MeSH Terms] OR “ultrasound”[All Fields] OR “ultrasonics”[MeSH Terms] OR “ultrasonics”[All Fields]))	1. ‘Plantar’/exp OR Plantar 2. ‘magnetic resonance imaging’/exp OR ‘magnetic resonance imaging’ 3. ‘MRI’/exp OR ‘MRI’ 4. ‘ultrasonography’/exp OR ultrasonography 5. ‘ultrasonics’/exp OR ultrasonics 6. ‘US’/exp OR ‘US’ 7. #2 OR #3 8. #4 OR #5 OR #6 9. #1 AND #7 AND #8

### Methodological quality assessment

Data were selected by two independent authors. The premier inclusion of studies was based on titles, abstracts, and keywords. Studies were included in the end when all authors agreed. Any differences or contradictions were resolved by discussion. The study quality was assessed by one author and independently verified by another author using the QUADAS form [13, 14] and following the guidelines provided in the Cochrane Handbook [15].

### Review process and statistical analysis

After included studies were collected, the software of the software Meta-DiSc version 1.4 (Unit of Clinical Biostatistics, Ramóny Cajal Hospital, Madrid, Spain.) and Review Manager 5.2 (The Nordic Cochrane Centre, The Cochrane Collaboration, 2012) was used for the statistical analysis. The analysis assessed sensitivity, specificity, positive likelihood ratios, and negative likelihood ratios for both MRI and US. For each analysis, 95% confidence intervals (CI) were adopted. Heterogeneity was tested by chi-square and Cochran-Q test. A *P* value of less than 0.05 was considered statistically significant. The fixed-effect model (Mantel-Haenszel test) and 95% CI were used initially. The random-effects model (DerSimonian-Laird test) was also considered to be used in which there was heterogeneity. Finally, summary receiver operating characteristic (sROC) plot was arranged to accesses the superior diagnostic accuracy for MRI and US.

## Result

**Fig. 1:**
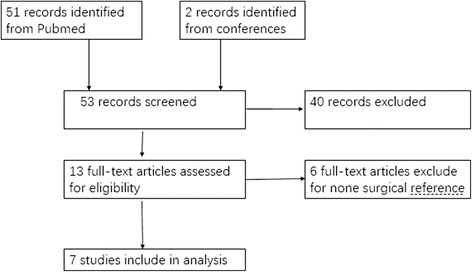
The flow chart shows how the articles were selected for eligible study

The search flow is shown in Fig. 1. A total of 53 papers were retrieved from the search strategy. After a review of the abstract and full texts, seven trials matched the inclusion criteria [10, 11, 16–20]. Two reported US for plantar plate tear [16, 17], four reported MRI for plantar plate tear [10, 18–20], and one [11] reported both US and MRI for plantar plate tear. All seven included studies were published in journals. A total of 246 samples were included (83 for US and 163 for MRI). The key characteristics of included studies are shown in Table 2.

**Table 2:** Characteristics of included studies

Studies	Number of samples	Index text	Age	Gender		Parameter of index text	Reference standard
				Male	Female		
Carlson et al. 2013 [16]	8	US	51.9(41–63)	0	8	Acuson Sequoia 512 Ultrasound Scanner (Siemens)	Surgery
Gregg et al. 2006 [11]	50	MRI and US	57(18–74)	N/A	N/A	MRI: 1.5-T MRI scanner (Signa Hi Speed Plus, General Electric Medical Systems) US: Antares scanner, (Siemens)with a high-frequency linear array probe (13-5VF; 11.4 MHz; dynamic range 60 dB; one focal zone)	Surgery
Klein et al. 2012 [10]	52	MRI	N/A	N/A	N/A	0.3 T extremity coil	Surgery
Klein et al. 2013 [17]	50	US	N/A	N/A	N/A	Sonosite M-turbo ultrasound and a linear 15-6 MHz transducer	Surgery
Nery et al. 2013 [18]	36	MRI	61(43–75)	8	20	1.0 to 1.5 T	Arthroscopy
Sung et al. 2012 [19]	45	MRI	52.1(28–70)	3	38	0.31 T (O-Scan Extremity MRI, Biosound Esaote, Indianapolis, IN)	Surgery
Yao et al. 1996 [20]	5	MRI	N/A	N/A	N/A	1.5 T (General Electric, Signa, Milwaukee, WI)	Surgery

The results for QUADAS methodological appraisal tool are shown in Table 3. The most primary limitation was uninterpretable/intermediate test results. It was unclear in all studies whether uninterpretable, indeterminate, or intermediate result was reported or not. The second frequent limit was the failure caused by the time period between index test and reference standard. When controlled, this removed the potential of a change in clinical status between investigations. The inclusion criteria were described in the methods of study, and the terms of inclusion were appropriately given to the nature of the pathology. Similarly, the inclusion criteria were also clearly documented between the individual studies. One study using MRI and one study using US which presented their imaging protocols insufficiently was not to allow its replication. The specific details of the surgical criteria were not clear among two studies.

**Table 3:** QUADAS appraisal tool results

study	1	2	3	4	5	6	7	8	9	10	11	12	13	14
Carlson 2013 [16]	Y	N	Y	U	Y	Y	Y	N	Y	Y	Y	Y	U	N/A
Gregg 2006 [11]	Y	Y	Y	U	Y	Y	Y	Y	Y	Y	Y	Y	N	N/A
Klein 2012 [10]	Y	Y	Y	U	Y	Y	Y	Y	Y	Y	Y	Y	N	N/A
Klein 2013 [17]	Y	Y	Y	U	Y	Y	Y	Y	Y	Y	Y	Y	N	N/A
Nery 2013 [18]	Y	Y	Y	U	Y	Y	Y	Y	Y	Y	Y	Y	N	N/A
Sung 2012 [19]	Y	Y	Y	U	Y	Y	Y	N	Y	Y	Y	Y	N	N/A
Yao 1996 [20]	Y	Y	N	U	N	Y	N	N	Y	Y	Y	Y	N	N/A

(1) Was the spectrum of patient representative of the patients who will receive the test in practice? (2) Were selection criteria clearly described? (3) Is the reference standard likely to correctly classify the target condition? (4) Is the time period between reference standard and index test short enough to be reasonably sure that the target condition did not change between the two tests? (5) Did the whole sample or a random selection of the sample receive verification using a reference standard of diagnosis? (6) Did patients receive the same reference standard regardless of the index test result? (7) Was the reference standard independent of the index test (i.e., the index test did not form part of the reference standard)? (8) Was the execution of the index test described in sufficient detail to permit replication of the test? (9) Was the execution of the reference standard described in sufficient detail to permit its replication? (10) Were the index test results interpreted without knowledge of the results of the reference standard? (11) Were the reference standard results interpreted without knowledge of the results of the index test? (12) Were the same clinical data available when test results were interpreted as would be available when the test is used in practice? (13) Were uninterpretable/intermediate test results reported? (14) Were withdrawals from the study explained?

**Fig. 2:**
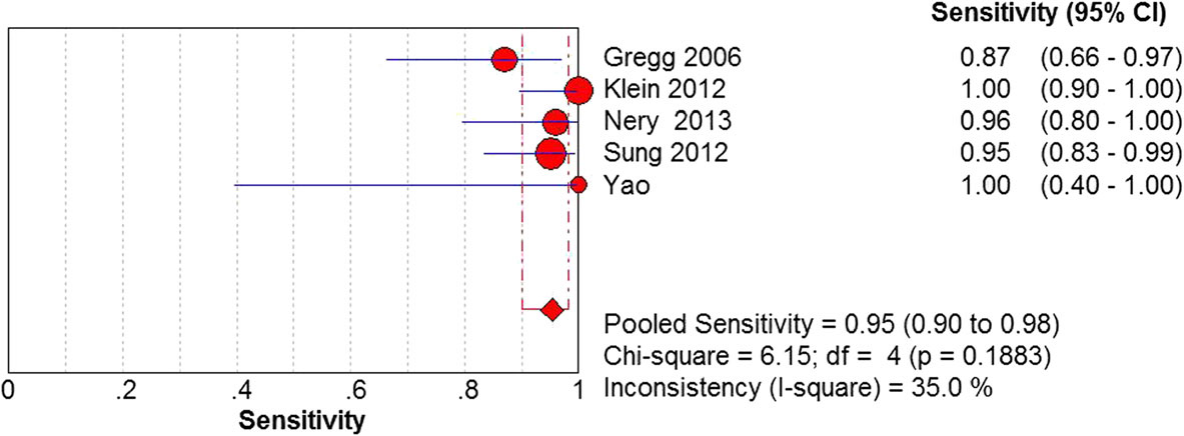
Forest plot depicting the sensitivity for the use of MRI in the detection of plantar plate tears

**Fig. 3:**
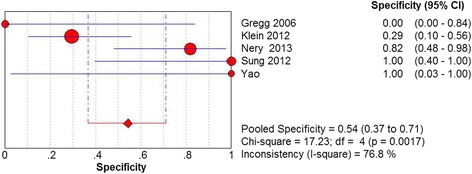
Forest plot depicting the specificity for the use of MRI in the detection of plantar plate tears

**Fig. 4:**
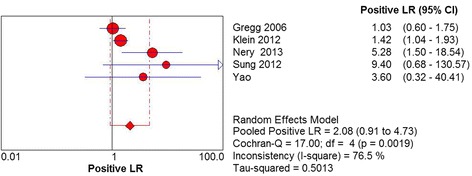
Forest plot depicting the positive likelihood ratio (PLR) for the use of MRI in the detection of plantar plate tears

**Fig. 5:**
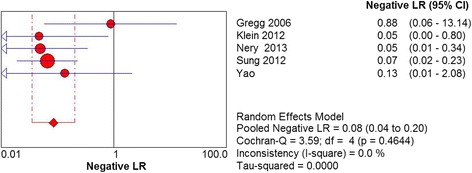
Forest plot depicting the negative likelihood ratio (NLR) for the use of MRI in the detection of plantar plate tears

Five studies, based on 163 samples, assessed the diagnostic test accuracy of MRI on tear of the plantar plate. The estimates for the sensitivity of MRI were 95% (95% CI 90 to 98%). No significant heterogeneity was detected among five studies (Chi^2^ = 6.15, degrees of freedom (*df*) = 4, *I* ^2^ = 35.0%, *P* = .1883) (Fig. 2). The specificity of MRI was analyzed for 54% (95% CI 37 to 71%), random effects model was used because of the existence of substantial heterogeneity (Chi^2^ = 17.23, *df* = 4, *I* ^2^ = 76.8%, *P* = .00017) (Fig. 3). The positive LR of MRI was estimated to be 2.08 (95% CI 0.91 to 4.73). The heterogeneity test indicated a statistical evidence of heterogeneity and we pooled data by a random effects model (Chi^2^ = 17.00, *df* = 4, *I* ^2^ = 76.5%, *P* = .0019) (Fig. 4). The test of the negative LR of MRI showed 0.08 (95% CI 0.04–0.20). No significant heterogeneity was detected among five studies (Chi^2^ = 3.48, *df* = 4, *I* ^2^ = 0.0%, *P* = .4644) (Fig. 5).

**Fig. 6:**
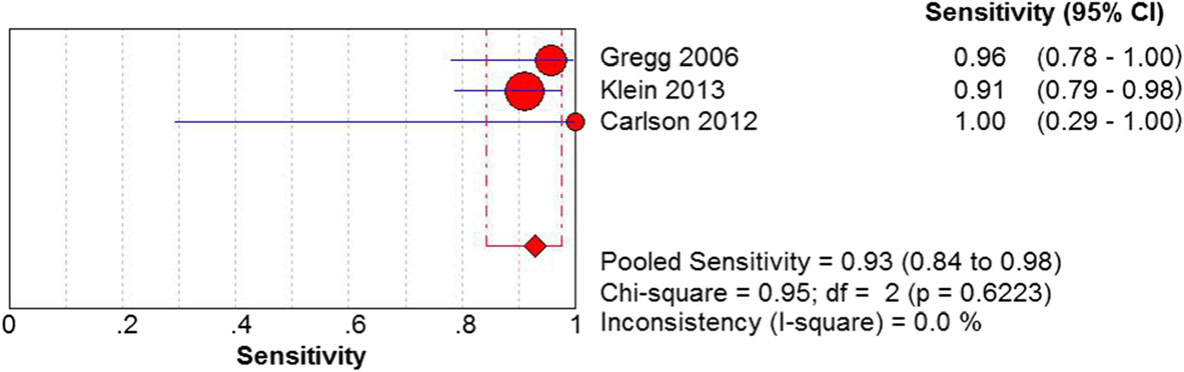
Forest plot depicting the sensitivity for the use of US in the detection of plantar plate tears

**Fig. 7:**
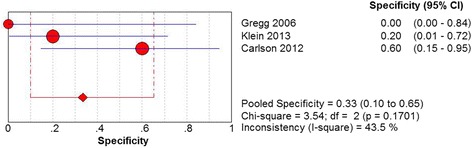
Forest plot depicting the specificity for the use of US in the detection of plantar plate tears

**Fig. 8:**
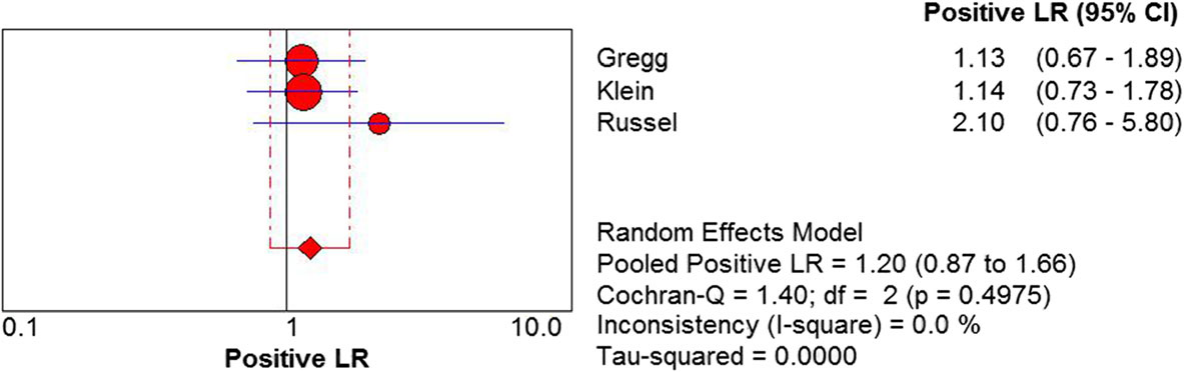
Forest plot depicting the positive likelihood ratio (PLR) for the use of US in the detection of plantar plate tears

**Fig. 9:**
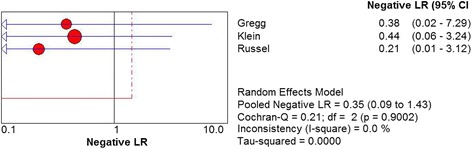
Forest plot depicting the negative likelihood ratio (NLR) for the use of MRI in the detection of plantar plate tears

Three studies including 83 patients with US assessed the diagnostic accuracy of plantar plate tear. The sensitivity of US was found to be 93% (95% CI 84 to 98%). Heterogeneity did not exist among three studies (Chi^2^ = 0.95, *df* = 2, *I* ^2^ < 0.01, *P* = .6223) (Fig. 6). It was estimated that the specificity of US was 33% (95% CI 10 to 65%). No significant heterogeneity was detected among three studies (Chi^2^ = 3.54, *df* = 2, *I* ^2^ = 43.5%, *P* = .1701) (Fig. 7). The positive LR of US was revealed as 1.20 (95% CI 0.87 to 1.66). No statistically significant heterogeneity was observed among three studies. (Chi^2^ = 1.40, *df* = 2, *I* ^2^ = 0.0%, *P* = .4975) (Fig. 8). The summary estimates for the negative LR of US was 0.35 (95% CI 0.09 to 1.43). No statistical heterogeneity was presented among three studies (Chi^2^ = 0.21, *df* = 2, *I* ^2^ = 0.0%, *P* = .9002) (Fig. 9).

**Fig. 10:**
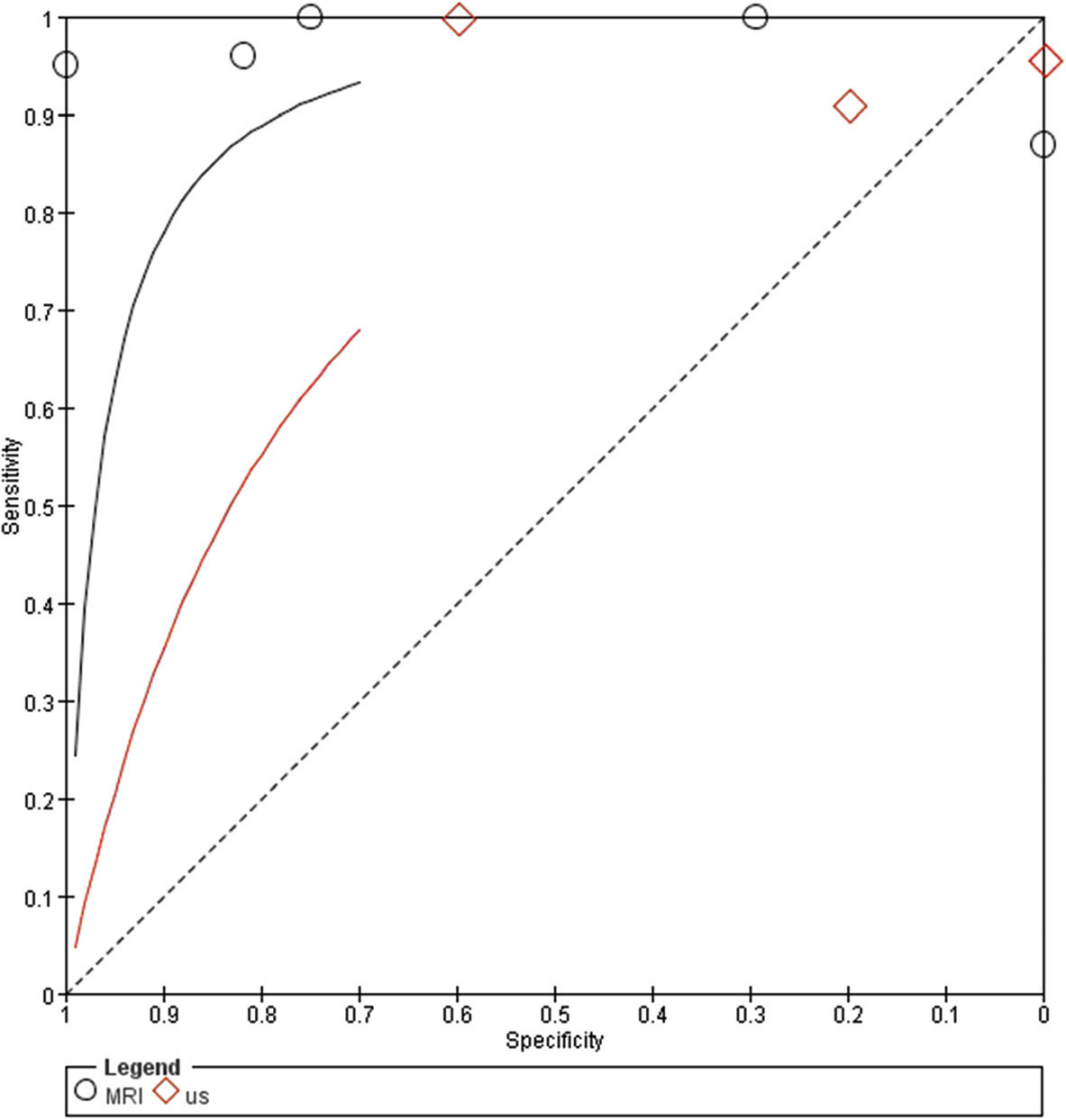
Summary receiver operator characteristic (sROC) comparing the diagnostic accuracy of MRI to US in the detection of plantar plate tears

As demonstrated in sROC, a better diagnostic accuracy for MRI was showed compared with US for the plantar plate tear diagnosis (Fig. 10).

## Discussion

The purpose of this study was to provide a better choice for detection of plantar plate tear. A similar rate was shown on sensitivities of MRI (95%) and US (93%) for assessment of plantar plate tear. US (33%) had lower specificity than MRI (55%) for plantar plate tears. Compared to US, MRI showed a higher positive LR (2.12 VS 1.20) and lower negative LR (0.08 VS 0.35). Based on the findings of this study, the results indicated that the MRI was superior to US for the detection of plantar plate tears in foot. It could also be concluded that MRI was superior to US on pathology.

The results of the QUADAS evaluation tool supported the suggestion that the current evidence-based study presented with a number of methodological limitations. One of the major limitations was uninterpretable test results. These problems were not often reported on studies for the accuracy of plantar plate tears, and the uninterpretable results were simply removed from studies. This may lead to bias of result for the test characteristics [13]. Besides, the studies poorly documented the duration between the reference and index tests. This is an important variable as pathology may be changing during the time elapsed between the reference and index tests. If there was a delay between reference and index tests, disease progression bias existed as misclassification. Each of these limitations should be considered in the future.

MRI was the most common method for detecting plantar plate. Yao firstly reported that MRI was thought to be better chosen compared to arthrography for assessment of plantar plate tears as a small local coil was used [11, 21]. MRI has been found both to be highly sensitive and to have specific modality for detecting of the plantar plate. In Sung’s study, MRI was found to have 96% accuracy, with 95% sensitivity, and 100% specificity [19]. In Klein’s study, MRI was found to be with 73.9% sensitivity and 100% specificity [10]. In our study, the sensitivity and specificity are 95 and 54%.

The use of diagnostic ultrasound in the identification of plantar plate tears has been increasingly reported [9, 10, 16, 17]. Ultrasound is an efficient choice to detect the presence of pathology of the plantar plate. In Klein’s study [17], ultrasound was found to be with 91.1% sensitivity and 25% specificity. However, a study showed that the positive rate of MTP drawer test in grade 1 MTP instability was found in 34.4% of joints with normal US finding [22]. In our study, the sensitivity and specificity of US are 93 and 33%.

Whether US can replace MRI for plantar plate tears was still debated. In Gregg’s standpoint [11], US is a dynamic examination that is inexpensive and safe, and is a viable alternative to MRI. Nonetheless, on Klein’s point [10], MRI is better able to detect plantar plate tear and localize pathology. The US is a less time consuming, and more comfortable detection, but US could not substitute MRI in all conditions. Based on our results, MRI was supported for the test of plantar plate tears because of MRI with lower negative LR, higher specificity, and positive LR and the same sensitivity than US.

The results of this diagnostic test accuracy study suggested that MRI was superior to US for detecting the plantar plate tears. However, the results of this study must also be interpreted attentively as the analyses demonstrated high *I* ^2^ values suggesting some statistical heterogeneity [23]. Possible reasons for this were that different magnetic field values (0.3–1.5 T) were used in different studies and/or experiences of the MRI radiologist in different hospitals were different.

## Conclusions

The available result suggests that MRI has better diagnostic accuracy for plantar plate tears than US. However, the concerns of risk for bias and heterogeneity should also be considered. High-quality trials are needed to assess the accuracy of MRI compared to US.

## Abbreviations

DF: Degrees of freedom
MRI: Magnetic resonance imaging
US: Ultrasound
